# Role of mucosal high-risk human papillomavirus types in head and neck cancers in Romania

**DOI:** 10.1371/journal.pone.0199663

**Published:** 2018-06-25

**Authors:** Ramona Gabriela Ursu, Mihai Danciu, Irene Alexandra Spiridon, Ruediger Ridder, Susanne Rehm, Fausto Maffini, Sandrine McKay-Chopin, Christine Carreira, Eric Lucas, Victor-Vlad Costan, Eugenia Popescu, Bogdan Cobzeanu, Nicolae Ghetu, Luminita Smaranda Iancu, Massimo Tommasino, Michael Pawlita, Dana Holzinger, Tarik Gheit

**Affiliations:** 1 University of Medicine and Pharmacy “Grigore T. Popa”, Discipline of Microbiology, Iași, Romania; 2 University of Medicine and Pharmacy “Grigore T. Popa”, Department of Pathology, Iași, Romania; 3 Roche MTM Laboratories, Mannheim, Germany; 4 Ventana Medical Systems, Inc., Tucson, Arizona, United States of America; 5 Department of Pathology, European Institute of Oncology, Milan, Italy; 6 Infections and Cancer Biology Group, International Agency for Research on Cancer, Lyon, France; 7 University of Medicine and Pharmacy “Grigore T. Popa”, Department of Oral and Maxillofacial Surgery, Iași, Romania; 8 University of Medicine and Pharmacy “Grigore T. Popa”, Department of Otorhinolaryngology, Iași, Romania; 9 University of Medicine and Pharmacy “Grigore T. Popa”, Department of Plastic surgery, Iași, Romania; 10 German Cancer Research Center (DKFZ), Division of Molecular Diagnostics of Oncogenic Infections, Heidelberg, Germany; Istituto Nazionale Tumori IRCCS Fondazione Pascale, ITALY

## Abstract

**Background:**

Limited information is available about the involvement of human papillomavirus (HPV) in head and neck squamous cell carcinomas (HNSCCs) in Romanian patients.

**Objective:**

To evaluate the HPV-attributable fraction in HNSCCs collected in Northeastern Romania.

**Materials and methods:**

In total, 189 formalin-fixed paraffin-embedded tissue samples (99 oral cavity tumors, 28 oropharynx, 48 pharynx, and 14 larynx/hypopharynx) were analyzed for HPV DNA and RNA using Luminex-based assays, and for overexpression of p16^INK4a^ (p16) by immunohistochemistry.

**Results:**

Of the 189 cases, 23 (12.2%) were HPV DNA-positive, comprising half of the oropharyngeal cases (14/28, 50.0%) and 9/161 (5.6%) of the non-oropharyngeal cases. HPV16 was the most prevalent HPV type (20/23, 86.9%), followed by HPV18 (5/23, 21.7%) and HPV39 (1/23, 4.3%). Only two (2/189, 1.1%) HNSCC cases were HPV-driven, i.e. positive for both HPV DNA and RNA.

**Conclusion:**

A very small subset of HNSCC cases within this cohort from Northeastern Romania appeared to be HPV-driven.

## Introduction

Head and neck squamous cell carcinoma (HNSCC) is the sixth most common cancer worldwide, with an estimated annual burden of 355,000 deaths and 633,000 incident cases [1]. Romania ranks second in mortality from HNSCCs in all-age males (32.4/100,000) among European countries [1].

HNSCCs are etiologically heterogeneous, being caused by tobacco use, alcohol consumption, poor oral hygiene, exposure to certain chemicals, and genetic features [2–4], as well as viral infections [5, 6]. High-risk (HR) human papillomavirus (HPV) infections have been associated with a subset of HNSCCs [7, 8]. HPV16 is the most common type, being present in more than 80% of HNSCCs [9, 10].

Chaturvedi et al. (2013) reported that the incidence of oropharyngeal cancer increased significantly in developed countries from 1983 to 2002 [11]. The proportion of HPV-positive oropharyngeal cancers among HNSCCs has been increasing over the past decades in many parts of the world, whereas the overall incidence of HNSCC is decreasing, consistent with declines in tobacco use [12]. Several studies reported a steady increase in the proportion of HPV-driven oropharyngeal cancer cases in the United States [13], in Sweden [14, 15], in Australia [16], and in New Zealand [17]. HPV has also been associated, to a much lesser extent, with non-oropharyngeal cancers such as oral or laryngeal cancer. In central India, less than 2% of these cancers were HPV-driven [18].

The prevalence of HPV DNA in HNSCCs varies greatly by study, cancer site, and geographical area [19, 20], being high in oropharyngeal cancer cases from the United States (71.0%) [21], eastern Denmark (62%) [22], and the Czech Republic (57.0%) [23], whereas several studies reported the absence of HPV DNA in oropharyngeal cancer cases from Mozambique [24] and China [25], or a low or intermediate HPV prevalence in Germany (34.4%) and Brazil (15.5%) [26]. All these studies are based on HPV DNA detection techniques. However, several independent studies have highlighted that the detection of HPV DNA alone is not sufficient to accurately define HPV-driven HNSCCs [18, 27–29]. The use of additional markers, such as viral RNA and p16^INK4a^ (p16) expression as a surrogate for HPV-induced transformation, allows a more precise classification of HNSCC.

In a recent study, the HPV-attributable fraction based on positivity for HPV DNA and for either HPV E6*I mRNA or p16, was 22.4%, 4.4%, and 3.5% for cancers of the oropharynx, oral cavity, and larynx, respectively [30]. Similar rates have been obtained in Kazakhstan, where 25.7% of oropharyngeal cancer cases tested positive for HPV DNA and p16 [31], and in Northeastern Italy, where 20% of oropharyngeal cancer cases tested positive for HPV DNA and HPV RNA [32]. In central India, HPV DNA/RNA double positivity was found in only 9.4% of oropharyngeal cancer cases [18]. HNSCCs from the Philippines all tested negative for both HPV DNA and HPV RNA [33]. In addition, in a recent study [30] based on 3680 HNSCCs from Europe, Africa, Asia, and the Americas, 22.4% of the oropharyngeal cancers tested positive for HPV DNA and for either HPV RNA or p16, and 18.5% were positive for all three markers. South America had the highest HPV-attributable fraction (53.6%) in oropharyngeal cancer, followed by Central and Eastern Europe (50.0%), Northern Europe (50.0%), Eastern Asia (22.4%), Central America (19.7%), Western Europe (19.4%), and Southern Europe (9.4%).

In Romania, limited information is available about the involvement of HPV in HNSCC. In this study, we aimed to determine the HPV-attributable fraction in HNSCC by analyzing HPV DNA and HPV RNA status, as well as by determining the p16 expression level, within a large retrospective cohort of HNSCC cases from Northeastern Romania.

## Materials and methods

### Patients and samples

Two hundred and three HNSCC patients were identified in the Departments of Oral and Maxillofacial Surgery, Otorhinolaryngology, and Plastic Surgery at the University of Medicine and Pharmacy “Grigore T. Popa” (Iași, Romania), from January 2010 to September 2014. All specimens were fixed for 18–24 hours in 10% neutral buffered formalin, at room temperature. The formalin-fixed, paraffin-embedded (FFPE) HNSCC blocks included squamous cell carcinoma of the oropharynx (International Classification of Diseases for Oncology [ICD-O] C01 –base of tongue, C02.4 –lingual tonsil, C09 –tonsil, C10 –oropharynx), pharynx (ICD-O C14 –other and ill-defined sites in the lip, oral cavity and pharynx, C14.8 –overlapping lesion of lip, oral cavity and pharynx), oral cavity (ICD-O: C00.0–C00.9, C01, C02.0–C02.9, C03.0–C03.9, C04.0–C04.9, C05.1–C05.9, C06.0–C06.9, C09.1–C09.9, C10), and hypopharynx and larynx (ICD-O: C13, C32). The FFPE tissue samples were retrieved from the hospital archives and comprised 34 HNSCC cases from the oropharynx and 169 HNSCC cases outside the oropharynx (16 larynx/hypopharynx, 51 pharynx, and 102 oral cavity cancer samples). All patients were diagnosed with keratinizing or non-keratinizing squamous cell carcinomas. Histological analyses on hematoxylin and eosin (H&E) stained slides were performed in order to confirm that all FFPE blocks contain cancer tissues. Clinical and epidemiological information was collected from the hospital databases using a form and questionnaire developed in the context of a European and Indian case study (HPV-AHEAD; http://hpv-ahead.iarc.fr). Ethical clearance for the investigations reported in this study was obtained from the Institutional Ethical Committee of the University of Medicine and Pharmacy “Grigore T. Popa”, Iași, Romania (reference number 7150). The study implied the use of archival material only, and it did not envisage any contact with the patients. Adequate measures to ensure data protection, confidentiality, patients’ privacy, and anonymization were taken into account. No informed consent was available, due to the retrospective design of the study and the large proportion of deceased and untraceable patients. All data were fully anonymized before access.

### Preparation of paraffin sections and DNA extraction

Each FFPE block was sectioned according to the HPV-AHEAD protocol, which includes the preparation of 31 sections from each FFPE tissue block. Sections 1, 10, and 31 (S1, S10, and S31) were used for histology, S2 and S9 were used for p16 immunohistochemistry (IHC), and S11–S30 were stored for future IHC analyses in independent studies. In addition, S3–S5 and S6–S8 were collected in two different vials and subsequently used for DNA and RNA analysis [34]. To minimize the risk of cross-contamination during sectioning, a new blade was used for each FFPE block and the microtome was extensively cleaned after each block with ethanol 70% and DNA Away (Dutscher, Brumath, France). In addition, to monitor possible cross-contamination during the sectioning, empty paraffin blocks were processed every 10th cancer specimen. DNA was extracted by an overnight incubation of the paraffin tissue sections in a digestion buffer (10 mM Tris/HCl pH 7.4, 0.5 mg/ml proteinase K, and 0.4% Tween 20) [35]. The percentage of tumor cells (0%, <10%, 10–50%, 50–90%, >90%) was estimated by two pathologists (MD, IAS) on H&E-stained slides (S1 and S10) [34].

### HPV DNA genotyping

HPV DNA positivity was determined by using a type-specific multiplex genotyping (TS-MPG) assay, which combines multiplex PCR and bead-based Luminex technology (Luminex Corporation, Austin, TX) as previously described [36, 37]. This assay detects 19 HR or probable high-risk (pHR) HPV types (HPV16, 18, 26, 31, 33, 35, 39, 45, 51, 52, 53, 56, 58, 59, 66, 68a and b, 70, 73, and 82) and two low-risk (LR) HPV types (HPV6 and 11), as well as cellular beta-globin gene, which is used to control for DNA quality. After PCR amplification, 10 μl of each reaction mixture was analyzed by multiplex HPV genotyping (MPG) using Luminex technology (Luminex Corporation, Austin, TX) as described previously [37, 38]. All HPV DNA-positive FFPE specimens and a randomly selected subgroup of approximately 10% of HPV DNA-negative specimens were further analyzed for the presence of HPV E6*I mRNA and for overexpression of the cell-cycle inhibitor p16, which is considered a surrogate marker for HPV infection. The 10% of HPV DNA-negative cases were selected randomly and blindly, while the study was still anonymized.

### HPV RNA analysis

Total RNA was purified from three pooled sections of the same tissue block using the Pure Link FFPE Total RNA Isolation Kit (Invitrogen, Carlsbad, CA) as described previously [27]. RT-PCR was carried out using the QuantiTect Virus Kit (Qiagen, Hilden, Germany), in a total volume of 25 μl containing 5 μl of 5xQuantiTect Virus Mastermix, 0.25 μl of 100xQuantiTect Virus RT Mix, 0.4 μM of each oligonucleotide, and 1 μl RNA as described previously [39]. The HPV type-specific E6*I mRNA assay developed for 20 HR- or pHR-HPV types [39] was applied for the detection of viral transcripts. The assay amplifies a 65–75 base pair amplicon of HPV and an 81 base pair amplicon of ubiquitin C (ubC) cDNA. Biotinylated amplification products are hybridized to ubC and HPV type-specific probes representing splice junction sequences on Luminex beads, followed by staining with streptavidin-phycoerythrin, and quantified in a Luminex analyzer. The use of a splice product sequence as detection probe makes this assay absolutely specific for RNA and avoids false positivity from residual viral DNA in the RNA preparation, which is a risk in RNA assays assessing unspliced RNA sequences.

The analytical sensitivity of the respective assays per reaction is 10,000 copies for HPV70, 1,000 copies for HPV67, and 10–100 copies for the remaining 19 HPV types and for ubC [39]. The HPV RNA assay has been widely applied and validated as a marker for HPV transformation in carcinoma of the anogenital region, such as the cervix [39, 40], vulva [41], penis [42], lung [43], and scrotum [44], as well as carcinoma of the head and neck [30, 33], and specifically oropharynx [45, 46], unknown primary of the neck [47, 48], larynx [27], and esophagus [49].

All HPV DNA-positive specimens and the randomly selected 10% of HPV DNA-negative specimens were analyzed for the presence of (i) HPV16 E6*I mRNA and (ii) ubC mRNA as a cellular mRNA positive control. Tissues positive for DNA of a non-HPV16 type were, in addition, analyzed for E6*I mRNA of the respective type. Specimens that were HPV E6*I and/or ubC mRNA-positive (RNA+) in RNA analysis were considered HPV RNA valid.

### p16 immunohistochemistry

Expression of p16 was evaluated manually by IHC in FFPE sections using the CINtec p16 Histology kit (Roche mtm laboratories AG, Mannheim, Germany) according to the instructions of the manufacturer. Briefly, slides were de-paraffinized in xylene and rehydrated in graded alcohol. The antigens were retrieved for 10 minutes using a pH 9.0 epitope retrieval solution (95–99 °C), followed by a 20 minute cool-down period at room temperature. Different from the instructions of the manufacturer, specimens were then microwaved in preheated Vector H-3300 unmasking solution (Vector Laboratories, Burlingame, CA) for 15 minutes. This step was followed by incubation of the p16 primary mouse anti-human antibody (clone E6H4) for 60 minutes. The samples were subsequently incubated with the goat anti-mouse IgG secondary antibody/peroxidase conjugate reagent, followed by signal generation using DAB. Finally, slides were counterstained with hematoxylin, dehydrated, mounted with permanent mounting medium, and cover-slipped. Immunoreactivity was visualized by light microscopy. Expression of p16 was evaluated by IHC in all HPV DNA-positive FFPE specimens and in a randomly selected subgroup of approximately 10% of HPV DNA-negative specimens. A continuous, diffuse staining for p16 within the cancer area of the tissue sections was considered as positive, and a focal staining or no staining was considered negative. Positive p16 expression was defined as diffuse nuclear and cytoplasmic staining in 70% or more of the tumor cells. The validity of the p16 IHC staining result was assessed by evaluating the presence of p16 internal control staining. IHC slides were evaluated by RR, FM, and DH blinded to any other clinical information or HPV DNA or RNA status, as specified in the HPV-AHEAD protocol [34]. Discrepant cases were re-checked by a pathologist, and the final classification of the staining was based on the majority consensus of the working group.

## Results

Of the 203 HNSCC cases, 2 cases (1 oral cavity and 1 pharyngeal) were excluded due to insufficient DNA quality as evidenced by negative ß-globin results, and 11 cases were excluded due to invalid RNA and/or p16 data. One case was excluded as the tissue block did not contain cancer tissue. The final study therefore comprised 189 HNSCC patients, with a median age of 62.5 years (range, 35–89 years). The vast majority of the patients were male: n = 171 (90.5%) (Table 1). Only FFPE blocks where the first and last H&E sections reflected tumor tissue were included in the study. More than 36% of the samples showed >50% of invasive carcinoma in the section, while 47.6% and 15.9% of the samples showed respectively 10–50%, and <10% of tumor cells.

**Table 1:** Description of Romanian HNSCC cases by HR-HPV DNA status.

Description	n	HPV DNA-positive n (%)	HPV DNA-negative n (%)
**Number of cases**	189	23 (12.2)	166 (87.8)
**Median age (range), years**	62.5 (35–89)	62 (43–86)	63 (35–89)
**Sex**			
Female	18	2 (11.1)	16 (88.9)
Male	171	21 (12.3)	150 (87.7)
**Cancer site**			
Oropharynx	28	14 (50.0)	14 (50.0)
Non-oropharynx	162	9 (5.6)	153 (94.4)
Oral cavity	99	4 (4.0)	95 (96.0)
Pharynx	48	0 (0.0)	48 (100)
Larynx*	14	5 (35.7)	9 (64.3)

Row percentages are shown; n, number

*includes hypopharyngeal cancer (n = 2)

Table 2 shows the HPV DNA, RNA, and p16 detection in HNSCC cases. HPV DNA was detected in 23 of the 189 (12.2%) HNSCC cases. HPV16 was the most prevalent type, being present in 20 of the 23 HPV DNA-positive tumors (86.9%), followed by HPV18 (5/23, 21.7%) and HPV39 (1/23, 4.3%). The oropharynx cases showed higher HPV DNA prevalence (14/28, 50.0%), followed by cancers of the larynx (5/14, 35.7%) and of the oral cavity (4/99, 4.0%). Multiple HPV type infections were detected in 3 HNSCC cases; 2 cases were positive for both HPV16 and HPV18 (1 larynx case and oropharynx case), and 1 oral cancer was positive for both HPV18 and HPV39 (Tables 1 and 2). One larynx case was positive for a low-risk HPV type (HPV6).

**Table 2:** HR-HPV DNA, RNA and p16 positivity in HNSCC subsites by HPV status.

		All HNSCC (N = 189)	Oropharynx (N = 28)	Non-oropharynx (N = 161)
HPV type	Marker positivity	Positive N (%)	Positive N (%)	Positive N (%)
**Any HR-HPV**	DNA	23 (12.2)	14 (50.0)	9 (5.6)
	DNA & RNA^‡^	2 (1.1)	1 (3.6)	1 (0.6)
	DNA, RNA & p16^‡^	0 (0.0)	0 (0.0)	0 (0.0)
**HPV16**	DNA	20 (10.6)	12 (42.9)	8 (5.0)
	DNA & RNA	1 (0.5)	0 (0.0)	1 (0.6)
	DNA, RNA & p16	0 (0.0)	0 (0.0)	0 (0.0)
**Non-HPV16 HR types**	DNA	5 (2.6)	3 (10.7)^1^	2 (1.2)^2^
	DNA & RNA	1 (0.5)	1 (3.6)^3^	0 (0.0)
	DNA, RNA & p16	0 (0.0)	0 (0.0)	0 (0.0)

^‡^HPV RNA and p16 expression was examined in all HR-HPV DNA-positive cases (n = 23) and a randomly selected subset of HR-HPV DNA-negative cases (n = 13). All HPV DNA-negative cases were RNA-negative. One case was p16-positive.

^1^Coinfection, HPV16 plus HPV18 (n = 1), and single infections, HPV18 (n = 2).

^2^Coinfections, HPV16 plus HPV18 (n = 1), and HPV18 plus HPV39 (n = 1)

^3^HPV18 (n = 1)

HPV RNA and p16 expression was examined in all HPV DNA-positive cases (n = 23) and a randomly selected subset of HPV DNA-negative cases (n = 13). The percentage of HPV-related HNSCCs was 1.1% (2/189) for both HPV DNA and RNA positivity. The highest percentage of combined HPV DNA and RNA positivity was found in the oropharynx (1 of the 28 HPV DNA-positive cases, 3.6%). The corresponding tonsil case tested positive for HPV18. Only one non-oropharyngeal case (1/161, 0.6%) was positive for both HPV DNA and RNA. The corresponding posterior hypopharyngeal wall case tested positive for HPV16 (Table 2).

The p16 IHC data were stratified per HR-HPV DNA and RNA status (Table 3). Only one HPV DNA-positive case (1/23; 4.3%) was p16-positive, and 7.7% (1/13) of HPV DNA- and RNA-negative cases were p16-positive, regardless of the anatomical sub-localization. In addition, none of the HNSCC cases that were HPV DNA- and RNA-positive tested positive by p16 IHC. Moreover, 2 of the 34 HPV RNA-negative cases (5.9%) were p16-positive (Table 3).

**Table 3:** p16 IHC data stratified per HR HPV DNA and RNA status.

**HPV type**	**Any HR-HPV DNA-positive**				Any HR-HPV DNA-negative*	
	(n = 23)				(n = 13)	
	Any RNA+		Any RNA-		Any RNA- (n = 13)	
	(n = 2)		(n = 21)			
	p16+	p16-	p16+	p16-	p16+	p16-
	n (%)	n (%)	n (%)	n (%)	n (%)	n (%)
HNSCC	0 (0)	2 (100)	1 (4.8)	20 (95.2)	1 (7.7)	12 (92.3)
**Subsite** Oropharynx	0 (0)	1 (50)	1 (4.8)	12 (57.1)	0 (0)	1 (7.7)
Non-oropharynx	0 (0)	1 (50)	0 (0)	8 (38.1)	1 (7.7)	11 (84.6)
**HPV type**	**HPV16 DNA-positive**				HPV16 DNA-negative*	
** **	(n = 20)				(n = 16)	
	HPV16 RNA+		HPV16 RNA-		HPV16 RNA- (n = 16)	
	(n = 1)		(n = 19)			
	p16+	p16-	p16+	p16-	p16+	p16-
	n (%)	n (%)	n (%)	n (%)	n (%)	n (%)
HNSCC	0 (0)	1 (100)	1 (5.3)	18 (94.7)	1 (6.3)	15 (93.8)
**Subsite** Oropharynx Non-oropharynx	0 (0)	0 (0)	1 (5.3)	0 (0)	0 (0)	3 (18.8)
	0 (0)	1 (100)	0 (0)	18 (94.7)	1 (6.3)	12 (75)
**HPV type**	**Other HR-HPV DNA-positive**				Other HR-HPV DNA-negative*	
** **	(n = 5)				(n = 31)	
	Other HR-HPV RNA+		Other HR-HPV RNA-		Other HR-HPV RNA-	
	(n = 1)		(n = 4)		(n = 31)	
	p16+	p16-	p16+	p16-	p16+	p16-
	n (%)	n(%)	n (%)	n(%)	n (%)	n(%)
HNSCC	0 (0)	1 (100)	0 (0)	4 (100)	2 (6.5)	29 (93.5)
**Subsite** Oropharynx Non-oropharynx	0 (0)	1 (100)	0 (0)	2 (50)	1 (3.2)	12 (38.7)
	0 (0)	0 (0)	0 (0)	2 (50)	1 (3.2)	17 (54.8)

*None of the HPV DNA-negative cases were RNA-positive; represents column percentages.

Among the cases that tested positive for HPV DNA, the smoking status was available for only 10 patients, among whom 8 were current smokers and 2 were former smokers. Most importantly, the clinical information was available for the HPV-driven HNSCCs (n = 2). Both tumors (1 tonsil case and posterior hypopharyngeal wall case) were late-stage (III and IV) and were from current smokers: patients aged 54 years (female) and 55 years (male), respectively.

## Discussion

It is now well demonstrated that mucosal HR-HPV types, mainly HPV16, are causally involved in a significant proportion of oropharyngeal cancers and to a much lesser extent in a subset of other HNSCCs [30]. However, the contribution of HR-HPV to the carcinogenesis of HNSCC appears to be subject to major geographical variability [10].

Compared with other European countries, cervical cancer rates are highest in Romania (28.6/100,000), highlighting the importance of HPV infections in this population. However, limited information is available about HPV-associated HNSCC in Romania [50]. Here, we have evaluated the contribution of HPV to HNSCC development in a study in Northeastern Romania by analyzing HPV DNA and HPV RNA status within a large retrospective cohort of HNSCC cases, as well as by determining the p16 expression status. The most frequent HR-HPV type was HPV16, followed by HPV18 and HPV39. The high HPV16 DNA prevalence in this study was similar to other findings published in the literature [51, 52].

Approximately 12% of HNSCCs tested positive for HPV DNA, but only 1.1% of all cases (n = 2) were positive for both HPV DNA and RNA and thus considered as being HPV-driven [18, 53–56]. According to a recent review [55], the term HPV-positive oropharyngeal squamous cell carcinoma (OPSCC) refers to carcinomas of the oropharynx presumed to be associated with HPV, on the basis of positivity to HPV DNA and p16 IHC. In this study, only one case tested positive for both markers: HPV DNA and p16. Thus, the fraction of HNSCCs attributable to transforming HPV infections in this Romanian region appeared to be considerably lower compared with various other geographical regions [10, 30, 57]. However, the low HPV prevalence in the FFPE samples is in line with the analysis of fresh tumor tissues from Romania, all being HPV-negative [58].

Both HPV DNA- and RNA-positive cases tested negative for p16. In addition, the great majority (95.6%) of HPV DNA-positive cases were p16-negative. The lack of expression of p16 in HPV DNA- and RNA-positive cases has been reported in other studies [18, 30]. These data contrast with the scenario observed in the Netherlands, where a double positivity for p16 and HPV DNA was shown to be valid to identify HPV RNA-positive cases [55, 57], and in Italy, where a fair agreement between HPV16 RNA-positivity and p16 overexpression in oropharyngeal cancer has been reported [59]. The absence of p16 expression in HPV DNA-positive HNSCC could be due to the fact that HPV, despite its presence in the tumor, is biologically inactive and is present in the tumor as a passenger virus or viral contaminant. Loss of p16 expression is a frequent event in cancer, and it occurs by deletion, point mutation, or hypermethylation [60–63]. The inactivation of p16 by hypermethylation of its promotor is common in HNSCC [64–66]. Hypermethylation of p16 promotor has been reported to be an early event in the development of oral cancer [67, 68]. Exposure to certain carcinogens, such as tobacco, may lead to alterations of p16 expression [18]. Indeed, hypermethylation of the p16 promoters was observed in several smoking-related human cancers, for example in non-small cell lung carcinoma [69–71] and cervical squamous cell carcinoma [72]. In addition, increased methylation of p16 was observed in laryngeal squamous cell carcinoma [73], and in normal oral mucosa [74], in smokers. Therefore, loss of p16 expression by hypermethylation in HNSCC, due to smoking or to other exposure factors [75, 76], could precede HPV infection, which would not induce p16 accumulation in this specific circumstance. Alternatively, Halec et al. [40] suggested that increasing chromosomal instability induced by HPV oncoproteins may lead to the loss of p16 in these cancers.

Moreover, one HPV DNA- and RNA-negative case tested positive for p16. A similar result was reported in a recent worldwide HNSCC study, thus suggesting that p16-positivity is not a perfect surrogate for HPV [30].

A limitation of our study is that information on other HNSCC risk factors (e.g. alcohol consumption, smoking) was available for only a few patients. This limitation was mainly due to the fact that the study implied the retrieval of archived HNSCC specimens, which were often not associated with detailed clinical information. From the available data in clinical questionnaires, 75.6% of the patients declared that they were smokers and 81.3% that they were users of alcohol. According to the latest WHO Report on the Global Tobacco Epidemic, 2015, the smoking prevalence in Romanian male adults was 37.4% [77]. This high percentage supports the idea that smoking can be an important risk factor for HNSCC in our study.

Tobacco smoking and alcohol consumption are important risk factors for HNSCC [12, 78]. More than 70% of HNSCCs are attributable to tobacco use and alcohol consumption [78]. Cigarette smoking is a strong risk factor for HNSCC independent of alcohol consumption [78]. The risk of developing laryngeal cancer was 10–20-fold higher in current smokers compared with non-smokers, and a 4–5-fold increased risk was observed for cancers of the oral cavity, oropharynx, and hypopharynx [79–81]. Alcohol consumption alone plays an independent role in approximately 4% of HNSCCs only [78]. However, pooled data from 17 case–control studies in Europe and the USA highlighted a multiplicative joint effect, rather than an additive effect, of tobacco use and alcohol consumption on HNSCC risk [82].

Another limitation of the study was the limited number of oropharyngeal cancers analyzed. This was mainly due to the fact that the majority of archival HNSCCs were from the oral cavity.

The results of this study warrant additional analyses, to describe the risk factors, the natural history and the clinical role of oral HPV infections in Romania.

In conclusion, a very small subset of HNSCC cases within this cohort from Northeastern Romania appeared to be HPV-driven, as evidenced by a low concordance between HPV DNA status and HPV RNA or p16 status of the analyzed HNSCC cases. Our study provides novel insights into the contribution of mucosal HR-HPV types in the development of HNSCC from Northeastern Romania and highlights potential differences in the carcinogenesis of HNSCC in this region compared with other European and non-European countries.
